# *Pogonophryne neyelovi*, a new species of Antarctic short-barbeled plunderfish (Perciformes, Notothenioidei, Artedidraconidae) from the deep Ross Sea

**DOI:** 10.3897/zookeys.296.4295

**Published:** 2013-04-30

**Authors:** Gennadiy A. Shandikov, Richard R. Eakin

**Affiliations:** 1 Department of Zoology and Animal Ecology, School of Biology, V.N. Karazin Kharkiv National University (KhNU), 4 Pl. Svobody, 61022 Kharkiv, Ukraine; 2 Southern Scientific-Research Institute of Marine Fisheries and Oceanography (YugNIRO), 2 ul. Sverdlova, 98300 Kerch, Crimea, Ukraine; 3 31223 Johnston Road, Guys Mills, Pennsylvania 16327, USA

**Keywords:** Southern Ocean, deep-water fishes, taxonomy, mental barbel, biology, reproduction

## Abstract

This paper continues descriptions of new deep-water Antarctic barbeled plunderfishes of the poorly known and the most speciose notothenioid genus *Pogonophryne*. It is based on a comprehensive collection obtained by the authors in 2009–2010 during an Antarctic toothfish (*Dissostichus mawsoni*) fishing trip. A new species, the hopbeard plunderfish *Pogonophryne neyelovi*, the twenty-second species of the genus, is described. The new species belongs to dorsally-spotted short-barbeled species forming the “*Pogonophryne mentella*” group. *Pogonophryne neyelovi* **sp. n.** is characterized by the following combination of characters: a very short and small mental barbel with an ovaloid and short terminal expansion covered by flattened scale-like processes that are mostly bluntly palmate; a moderately protruding lower jaw; a high second dorsal fin almost uniformly black and lacking a sharply elevated anterior lobe; pectoral fins striped anteriorly and uniformly light posteriorly; the anal and pelvic fins light; the dorsal surface of the head and the area anterior to the first dorsal fin covered with large, irregular dark brown blotches and spots; the ventral surface of the head, breast and belly without sharp dark markings. The new species is compared to the closest species *Pogonophryne brevibarbata*, *Pogonophryne tronio*, and *Pogonophryne ventrimaculata*. English vernacular names are proposed for all species of the genus.

## Introduction

Antarctic barbeled plunderfishes of the genus *Pogonophryne* (Perciformes: Notothenioidei: Artedidraconidae), the most speciose among notothenioid fish with a circum-Antarctic distribution, inhabit inshore and bathyal bottom zones of the Southern Ocean. *Pogonophryne* is one of the most taxonomically complex notothenioid genera (Eakin et al. 2001; Shandikov et al. 2013). Identification of *Pogonophryne* species is furher complicated by the deficiency of molecular-genetic data, known to date for only half (11) of the species (Eakin et al. 2009), and by the poor knowledge of intra- and interspecific variation due to the lack of material so far available for a comparative study. Among 21 species currently recognized within the genus *Pogonophryne* (Shandikov et al. 2013), whose limits are yet to be determined, 12 species (about 57%) belong to the most species-rich “*Pogonophryne mentella*” group of dorsally spotted species defined by Balushkin and Eakin (1998). Three new deep-water fishes belonging to the short-barbeled and medium-barbeled species of this group were recently described from the marginal seas of continental Antarctica (Eakin et al. 2008; Balushkin et al. 2010; Shandikov et al. 2013) − spotless nape plunderfish *Pogonophryne bellingshausenensis* Eakin, Eastman & Matallanas, 2008, shortbeard plunderfish *Pogonophryne brevibarbata* Balushkin, Petrov & Prutko, 2010 and turquoise plunderfish *Pogonophryne tronio* Shandikov, Eakin & Usachev, 2013. One more, the forth short-barbeled species − spotbelly plunderfish *Pogonophryne ventrimaculata* Eakin, 1987, is known only from the shelf waters of East Antarctica.

In addition to the currently recognized species of *Pogonophryne*, including the new species *Pogonophryne neyelovi*, seven previously unknown forms from the deep Ross and Amundsen seas that may represent formally undescribed species were considered by Shandikov (2013) as *Pogonophryne* species A to G in his brief review of the genus.

In this paper we continue descriptions of new species of the genus *Pogonophryne* collected as by-catch of Antarctic toothfish, *Dissostichus mawsoni* Norman, 1937 (Nototheniidae), by the first author as a CCAMLR (Commission for the Conservation of Antarctic Marine Living Resources) international scientific observer from Ukraine during the austral summer 2009–2010 fishing cruise of longliner F/V *Tronio* (Grupo Regal, Celeiro, Lugo, Spain) to the Ross and Amundsen seas. The twenty-second currently recognized species of the genus, the hopbeard plunderfish *Pogonophryne neyelovi* sp. n., is described herein from the deep Ross Sea.

## Materials and methods

Description of coloration was taken from fresh and formalin-preserved (10%) specimens, counts and measurements were taken from the formalin-preserved holotype following Shandikov et al. (2013) with digital callipers to the nearest 0.1 mm. Stage of gonad maturity (SGM, stages I to VI) was identified following Shandikov and Faleeva (1992) by visual examination with stereo-binocular microscope. Hepatosomatic index and gonadosomatic index (GSI) were calculated using body weight without swallowed bait.

Additional morphological data obtained by the authors from direct field observations and laboratory examination of specimens of most of the other species of the genus *Pogonophryne* were also used for the comparison, except for two species: finespotted plunderfish *Pogonophryne permitini* Andriashev, 1967 and longbeard plunderfish *Pogonophryne mentella* Andriashev, 1967, that were compared based on the original descriptions by Andriashev (1967).

Photos and drawings by G.A. Shandikov, except where otherwise indicated.

Abbreviations used: LACM, Natural History Museum of Los Angeles County, Los Angeles, California, U.S.A.; MNKhNU, Museum of Nature at V.N. Karazin Kharkiv National University, Ukraine; NMNZ, Museum of New Zealand Te Papa Tongarewa (National Museum of New Zealand), Wellington; SAIAB, South African Institute for Aquatic Biodiversity (formely J.L.B. Smith Institute of Ichthyology), Grahamstown, South Africa; UAB, Universidad Autónoma de Barcelona, Spain; USNM, Smithsonian InstitutionNational Museum of Natural History, Washington, D.C., USA; YPM, Yale Peabody Museum of Natural History, Yale University, USA; ZIN, Zoological Institute, Russian Academy of Sciences, St Petersburg, Russia; ZMH, Zoological Museum collections of Hamburg University, Germany.

## Results

### Pogonophryne neyelovi sp. n.

urn:lsid:zoobank.org:act:9D86C983-9211-495F-AB32-DFD2304FE055

http://species-id.net/wiki/Pogonophryne_neyelovi

**Figure 1.**
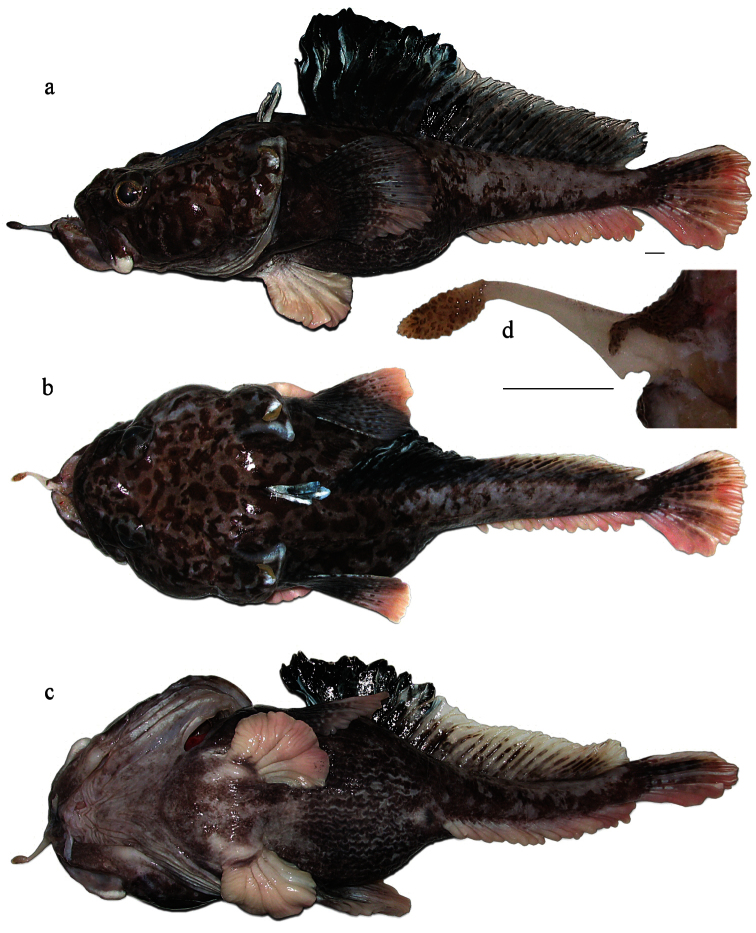
*Pogonophryne neyelovi* sp. n., holotype (MNKhNU R299), freshly caught male, 355 mm TL, 295 mm SL: **a** lateral view **b** dorsal view **c** ventral view **d** mental barbel (dorsal view): stem damaged by hook with removed flap of skin. Scale bars 10 mm.

Figures 1 2b 3 4 5c Table 1

#### Holotype.

MNKhNU R299 (Fig. 1), post-spawning male, 355 mm TL, 295 mm SL, weight (in formalin) 685 g, F/V *Tronio*, bottom Spanish long-line, set no. 49, Ross Sea (72°50.5'S, 176°49.2'E), depth 1,337 m, 12 January 2010, coll. G.A. Shandikov. Damage made by releasing the hook: symphysis of lower jaw disjointed and skin removed from stem of mental barbel though preserved in good condition.

#### Additional material.

Freshly caught female (not preserved, Fig. 3), 350 mm TL, 294 mm SL, F/V *Tronio*, bottom Spanish long-line, set no. 46, Ross Sea (72°50.1'S, 176°50.8'E), depth 1,350 m, 18 January 2010, coll. G.A. Shandikov. Freshly caught specimen, sex undetermined, probably female (not preserved, identified by photos, Fig. 4), 305 mm TL, 253 mm SL, F/V *Joong Woo 3*, bottom trot-line, set no. 43, Ross Sea (73°14.6'S, 177°52.4'W), depth 700–1,390 m, 13 January 2012, coll. Yu. Korzun.

#### Diagnosis.

A new species of the genus *Pogonophryne* distinguished from other species of the “*Pogonophryne mentella*” group by the following combination of characters. Short and tiny mental barbel, light-brownish dorsally, reaching anterior edge of orbit over snout with mouth closed; its length about 9% SL (Table 1). Very short poorly developed ovaloid terminal expansion composed of bicolored, white and brownish, scale-like and mostly bluntly palmate processes; its length less than 1/3 of barbel length. Lower jaw moderately protruding with weakly visible anterior teeth on symphysis and hidden dorsum of tongue when mouth closed. Second dorsal fin high in male (its height about 25% SL), lacking any prominent anterior elevated lobe, with longest rays (1^st^ to 7^th^) very soft, sinuous distally and branched from about mid-length; fin almost entirely black in coloration with bluish insertions in anterior one third and dark basally with light upper margin posteriorly. Dorsal surface of head and area anterior to first dorsal fin covered by dark-brown irregular spots, vermiculations and markings; belly, breast and lower surface of head brown or brownish in general, without distinct dark spots. Mandibular oral valve light. Pectoral fin vertically dark-striped in its anterior part and uniformly lightish in posterior part.

#### Description of holotype.

For all measurements and counts see Table 1. Body robust and tadpole-like from dorsal and ventral views. Head depth (maximum body depth) 20%  SL. First dorsal fin with two soft spines, comparatively low, length of its base very short. Second dorsal fin with 27 rays and very high, its dorsal profile not concave and without a prominent elevated anterior lobe. Longest second dorsal fin rays (1^st^ to 7^th^) very soft, sinuous distally and branched from about mid-length. Anal fin with 18 fleshy rays. Left and right pectoral fins with 20 rays. Pelvic fin not long, fleshy and notably wide (almost as wide as long). Posterior margin of caudal fin sharply rounded.

Eye filling orbit, snout rounded in dorsal view. Lower jaw moderately protruding (distance between tip of lower jaw and tip of upper jaw 1.9% SL or 4.8% of head length) with weakly visible anterior teeth on symphysis and hidden top of tongue when mouth closed. Posttemporal ridges not prominent. Both oral valves anteriorly possess sparsely distributed short and simple papillae; inner margins of both lips fringed.

**Figure 2.**
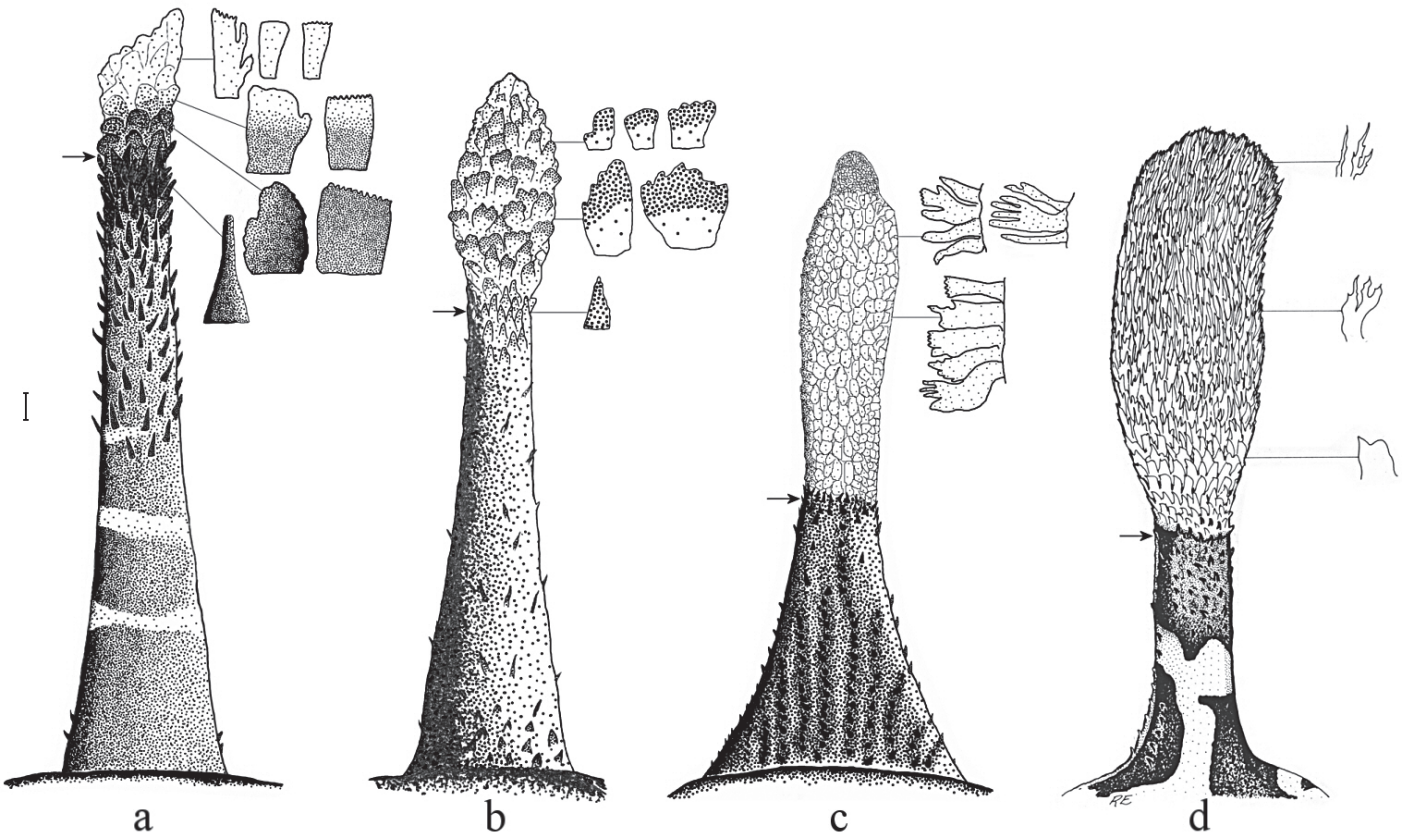
Mental barbel in short-barbeled species of the “*Pogonophryne mentella*” group, dorsal view, showing processes from various regions of terminal expansion; arrow indicates upper border of terminal expansion: **a** *Pogonophryne tronio*, holotype (MKhNU R295, formalin-preserved), male, 295 mm SL **b** *Pogonophryne neyelovi* sp. n., holotype (formalin-preserved) **c** *Pogonophryne brevibarbata*, female (MKhNU R298, just caught specimen), 262 mm SL **d** *Pogonophryne ventrimaculata*, holotype ZMH 46-1985, alcohol-preserved (from Eakin 1987: fig. 4), female, 214 mm SL. Scale bar 1 mm.

*Mental barbel* (Fig. 2b) small and short, its length about 9% of head length, somewhat rounded distally, reaching anterior edge of orbit in backward extended mode over snout with mouth closed. Barbel base about 21% as wide as long. Proximal part of stem covered by sparsely distributed short papillae; median part of stem covered by sparsely distributed elongated papillae; distal part of stem covered by densely distributed flattened and acute-angled processes. Terminal expansion short, ovaloid and barely wider than stalk (width about 3.2 mm or 12% of barbel length, depth about 2.8 mm or 10% of barbel length), composed of scale-like, mostly bluntly palmate (resembling cat’s paw) processes; bifurcated, truncate and serrated processes also occur.

*Teeth* on both jaws sharp and conical, slightly curved inwards, somewhat enlarged on tips of jaws in posterior rows, somewhat larger on upper jaw; up to five irregular rows at symphyses of lower and upper jaws.

*Gill rakers* short, denticulate, slightly laterally compressed or conical; in total 19 rakers on first arch: 1+0+9=10 in anterior series and 0+1+8=9 in posterior series; rakers on lower part of gill-arch distributed along entire length of ceratobranchial bone.

*Lateral line system*. Upper lateral line with 25 (left) and 26 (right) pores (tubular scales), middle lateral line with about 14 (left) and 15 (right) pores and tubular scales. Number of pores in cephalic sensory canals: preoperculo-mandibular 9/9; infraorbital 7/7; supraorbital 4/4, with 2/2 nasal and 2/2 interorbital pores; coronal commissure with 1 central pore; temporal 6/6; supratemporal 2, disrupted at middle, with 1 left and 1 right pores.

*Radiograph*. Total vertebrae 38: 16 abdominal and 22 caudal.

*Coloration*. In live specimen general ground coloration of upper and lateral surfaces of head, upper jaw, back and lateral trunk sandy-pale. Bases of pectoral fins uniformly dark-brown. Abdominal surface of body lacking evident dark spots or markings: belly generally dark brown with some lighter indistinct areas and longitudinal zigzag-like striations, Breast lighter than belly, lower surface of head light. Dorsal and lateral surface of head anterior to first dorsal fin, back and lateral parts of trunk covered by dark-brown, specific irregular (not rounded) markings − vermiculations and spots, markings covering up to 50% of upper surface of head anterior to first dorsal fin. Spotted skin extends over dorsal parts of eyes. Nostrils unpigmented. Upper jaw mostly spotted, posterior tips of maxillaries and angle of mouth uniformly white. Mandibular oral valve whitish to somewhat light brownish, maxillar oral valve whitish. Fringes on inner margins of both lips and oral cavity overall whitish. Mental barbel indistinctly bicolored dorsally: with brownish stem and bicolored, whitish and brownish, scale-like processes on terminal expansion, ventral surface of stem lighter.

Soft spines in first dorsal fin whitish, fin-fold blackish basally and light-bluish distally. Second dorsal fin generally black in anterior third with partly bluish fin-fold sectors along first to third and sixth-seventh rays, and dark basally with light upper margin posteriorly. Anal and pelvic fins light, somewhat pinkish (fleshy), without the contrasting (dark-light) vertical bicolored pigmentation typical of most congeners. Pectoral fins with about six to seven narrow dark vertical stripes anteriorly, distal third of fins light. Caudal fin with creamy-pinkish (fleshy) ground coloration, dark very basally and in median superior part more evident in three upper branched rays, with about six indistinct dark vertical stripes medially.

Gill chamber light, somewhat light-brownish. Peritoneum creamy white, with very sparsely distributed black dots.

In formalin-preserved specimen general features of pigmentation remain unchanged except for bluish and pinkish coloration on fins being faded.

#### Variability.

The second dorsal fin in the adult male is considerably higher than in the female (24.8% SL *vs*. 18.9% SL in female, Table 1), this may be due sexual dimorphism. In the female the point of primary branching of the anterior longest rays in the second dorsal fin is located somewhat above the mid-point of the rays (*vs*. mid-point of rays in male). General body and fin coloration is similar in both sexes. Wide, very fleshy and uniformly light pinkish pelvic fins, as well as fleshy anal-fin rays and uniformly light pinkish coloration of the anal fin and ground coloration of the caudal fin, may be considered as nuptial or age-related changes in post-spawning adults, both male and female. Similar nuptial or age-related changes in adult fish were recently found in *Pogonophryne tronio* by Shandikov et al. (2013). Immature subadult or juvenile individuals probably possess vertically bicolored (dark and whitish) anal and pelvic fins, and the pelvic fins are narrowed as can be seen in the smaller unpreserved specimen 305 mm TL (Fig. 4).

#### Distribution.

*Pogonophryne neyelovi* is a deep-water benthic species known from three captures in the Ross Sea at depths of 700–1,390 m.

#### Mode of life.

Like other morphologically close deep-water congeners (Shandikov et al. 2013) *Pogonophryne neyelovi* might be considered predatory in its feeding behavior. It probably also feeds by necrophagy. Three known specimens of *Pogonophryne neyelovi* were captured by hooks baited with large pieces (about 4×3×2 cm) of giant Peruvian squid, *Dosidicus gigas*. In the holotype, the liver is large, filling about 35% of the abdominal cavity length and creamy in coloration; hepatosomatic index reaches 2.0. Spawning probably took place within one month prior to the capture, around December – January. The testes are paired, well developed and folded, reaching about 68 mm in length, 14 mm in width and 6.5 mm in depth. GSI was about 0.6, and SGM apparently corresponds to early post-spawning stage VI or VI−II.

#### Etymology.

The new species is named after Alexey V. Neyelov who contributed significantly to the knowledge of Antarctic fishes, and to whom the first author is sincerely thankful for the valuable help during his PhD studies in Zoological Institute, Russian Academy of Scienses, St Petersburg, Russia. Vernacular names proposed for *Pogonophryne neyelovi*: hopbeard plunderfish – in English and хмельовуса бородатка [khmelyovusa borodatka] – in Ukrainian.

**Table:** **Table 1.** Measurements and counts for four short-barbeled plunderfishes of the “*Pogonophryne mentella*” group of the genus *Pogonophryne*

**Character**	***Pogonophryne neyelovi* sp. n.**		***Pogonophryne tronio***	***Pogonophryne brevibarbata***	***Pogonophryne ventrimaculata***
	**Ross Sea: holotype, male**	**Ross Sea: additional material, female**	**Ross Sea: from Shandikov et al. (2013), female and 2 males**	**Ross Sea: from Balushkin et al. (2010) and Shandikov et al. (2013), 2 females and male**	**Weddell Sea: from Eakin (1987), 3 females and 2 males**
TL, mm	355	350	280–315	267–325	–
SL, mm	295	294	230–260	219–262	115–214
SL, % TL	83.1	84.0	80.7–82.4	80.6–84.4	–
Weight, g	685	about 700	250–440	?–387	–
Depth of capture, m	1,337	1,350	900–1,225	1,036-1,163	248–419
Body (head) depth at posttemporal ridges, % SL	20.4	–	15.8–22.6	16.4–22.7	18.4–21.9
Body depth at origin of anal fin, % SL	13.4	–	12.2–14.4	12.2–14.8	11.4–13.8
Depth of caudal peduncle, % SL	5.6	–	5.4–6.3	–	5.2–5.9
Body (head) width at opercles, % SL	31.1	–	29.8–34.8	13.9	–
Body width at origin of anal fin base, % SL	13.1	–	10.1–11.2	9.0	–
First predorsal distance, % SL	32.3	–	31.7–32.8	32.1–36.1	34.2–35.7
Second predorsal distance, % SL	41.8	–	39.5–43.3	41.7–46.1	41.1–43.1
Postdorsal distance, % SL	8.2	–	6.3–8.4	7.2	–
Prepectoral distance, % SL	39.7	–	37.5–42.1	38.7	–
Prepelvic distance, % SL	31.9	–	29.7–32.8	31.4	–
Preanal distance, % SL	64.1	–	62.2–65.8	64.9–66.7	–
Length of caudal peduncle, % SL	6.6	–	6.1–6.7	2.4–6.5	5.4–6.1
Length of pectoral fin, % SL	23.5	–	24.4–26.8	23.5–27.4	24.3–26.7
Length of pelvic fin, % SL	18.6	17.8	17.2–20.3	15.4–17.4	17.3–20.0
Width of pelvic fin, % SL	16.9	12.6	9.7–18.2	8.0	–
Width of pelvic fin, % length of pelvic fin	about 90.9	about 70.9	about 52.5–89.7	about 52.0–60.0	–
Length of base of fist dorsal fin, % SL	2.4	–	1.7–2.2	1.8	–
Height of first dorsal fin, % SL	10.0	8.6	7.7–9.7	5.8–8.7	5.4–6.1
Height of first dorsal fin, % height of second dorsal fin	40.2	45.5	51.2–53.8	40.0–58.8	–
Length of base of second dorsal fin, % SL	51.5	–	50.3–51.4	52.6	–
Height of second dorsal fin, % SL	24.8	18.9	15.5–18.1	13.7–20.5	14.8–21.3
Height of posterior part of second dorsal fin, % SL	14.1	11.6	12.5–14.7	11.6	–
Height of posterior part of second dorsal fin, % height of second dorsal fin	56.6	61.1	80.9–90.5	84.7	–
Interdorsal distance, % SL	7.6	–	5.7–9.0	7.3–8.7	–
Length of anal-fin base, % SL	31.0	–	29.2–30.6	28.7	–
Height of anal fin, % SL	8.1	–	9.0–10.0	9.3	–
Length of caudal fin, % SL	18.4	–	19.8–21.2	20.2	21.5–24.8
Head length, % SL	38.0	38.6	37.1–39.4	37.3–42.0	39.3–42.2
Head depth at middle of eye, % SL	14.9	–	12.8–15.5	13.6	–
Head depth through middle of eye, % head length	39.3	–	34.5–40.3	36.3	–
Head (body) depth at posttemporal ridges, % head length	53.7	–	42.6–57.7	44.0–61.8	–
Head width at preopercles, % SL	29.5	–	27.9–33.6	29.9–35.0	25.6–35.7
Head width at preopercles, % head length	77.5	–	75.2–87.5	75.0–87.2	–
Head (body) width at opercles, % head length	81.8	–	80.2–90.3	85.7	–
Mental barbel length, % SL	9.1	8.5	9.6–11.6	8.3–11.8	11.2–12.9
Mental barbel length, % head length	23.9	22.1	25.1–30.6	22.3–29.4	–
Mental barbel expansion length, % mental barbel length	30.6	about 20	18.4–28.8	50.9–68.3	50.0–65.7
Mental barbel expansion width, % mental barbel length	about 12	–	[inconspicuous]	[inconspicuous]	twice wider than stem least width
Mental barbel base width, % mental barbel length	20.6	–	20.6–25.2	–	–
Mental barbel stem least width, % mental barbel length	about 10	–	6–8	–	–
Snout length, % SL	8.3	–	9.0–9.8	9.2–10.9	9.1–10.1
Snout length, % head length	21.9	–	24.1–25.0	23.5–26.1	–
Snout width, % SL	14.9	–	14.7–16.8	15.4	–
Snout width, % head length	39.3	–	39.7–43.2	41.3	–
Horizontal diameter of orbit, % SL	8.6	–	7.5–8.0	7.5–9.0	7.2–9.6
Horizontal diameter of orbit, % head length	22.6	–	20.1–20.5	20.2–25.0	–
Postorbital distance, % SL	22.6	–	21.7–23.8	22.4	22.6–25.2
Postorbital distance, % head length	59.4	–	58.7–62.1	60.0	–
Internostril distance, % SL	8.9	–	8.9–9.1	7.9–10.2	8.3–9.4
Internostril distance, % head length	23.3	–	23.0–23.9	21.3–25.5	–
Interorbital distance, % SL	7.4	–	6.7–7.3	6.8–7.3	6.1–6.5
Interorbital distance, % head length	19.5	–	17.5–19.7	16.3–19.7	–
Upper jaw length, % SL	18.0	–	16.7–17.3	15.9–19.7	14.9–17.1
Upper jaw length, % head length	47.4	–	43.4–45.1	42.7–49.0	–
Lower jaw length, % SL	21.3	–	18.3–21.5	19.0–20.7	–
Lower jaw length, % head length	56.0	–	49.1–56.0	49.2–51.5	–
**Counts:**					
First dorsal fin soft spines	2	2	2	2	2
Second dorsal fin rays	27	27	27–28	27–28	27–28
Anal fin rays	18	17	17–18	17–18	17
Pectoral fin rays	20/20	20/21	19–20	19–21	20–21
Vertebrae	16+22=38	–	16+22=38	16+21=37	(15–16)+(20+21)=36–37
Gill rakers, anterior row	1+0+9=10	2+0+9=11	(1–2)+0+(8–10)=9–10	1+0+(7+8)=8–9	6–10
Gill rakers, posterior row	0+1+8=9	0+1+7=8	(0–1)+(0–1)+(6–8)=7–9	(0–1)+(0+1)+(6–7)=7–8	7–8
Gill rakers, total	19	19	17–21	16	13–17
Upper lateral line pores	25/26	–	25–27	24–29	21–26
Middle lateral line pores	14/15	–	12–16	12–19	9–14

**Figure 3.**
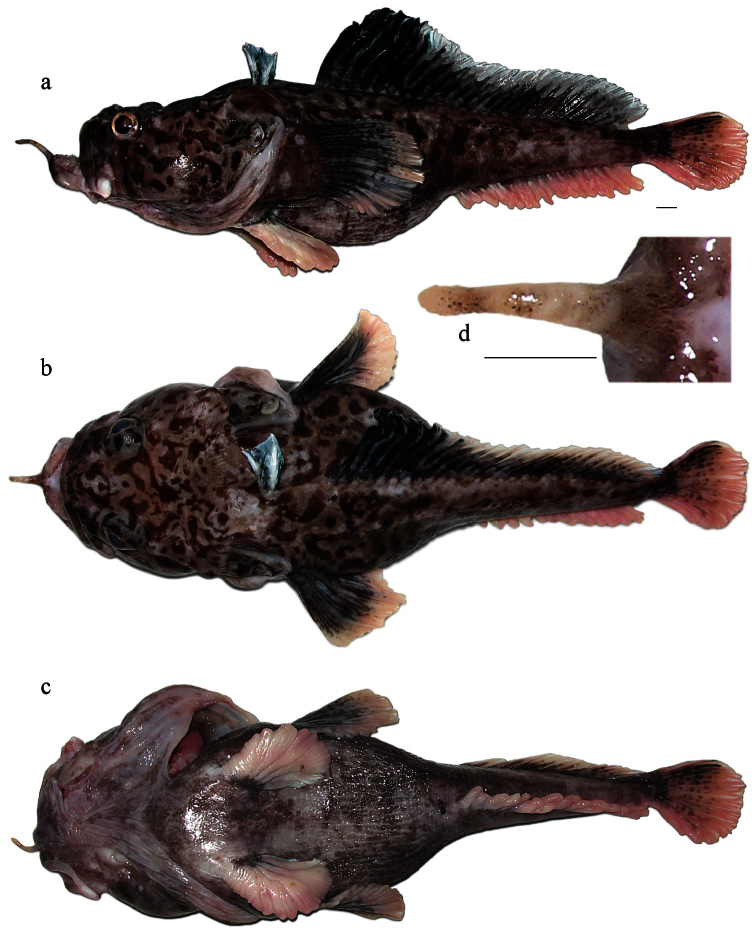
*Pogonophryne neyelovi* sp. n., freshly caught female (not preserved), 350 mm TL, 294 mm SL: **a** lateral view **b** dorsal view **c** ventral view **d** mental barbel (dorsal view). Scale bars 10 mm.

**Figure 4.**
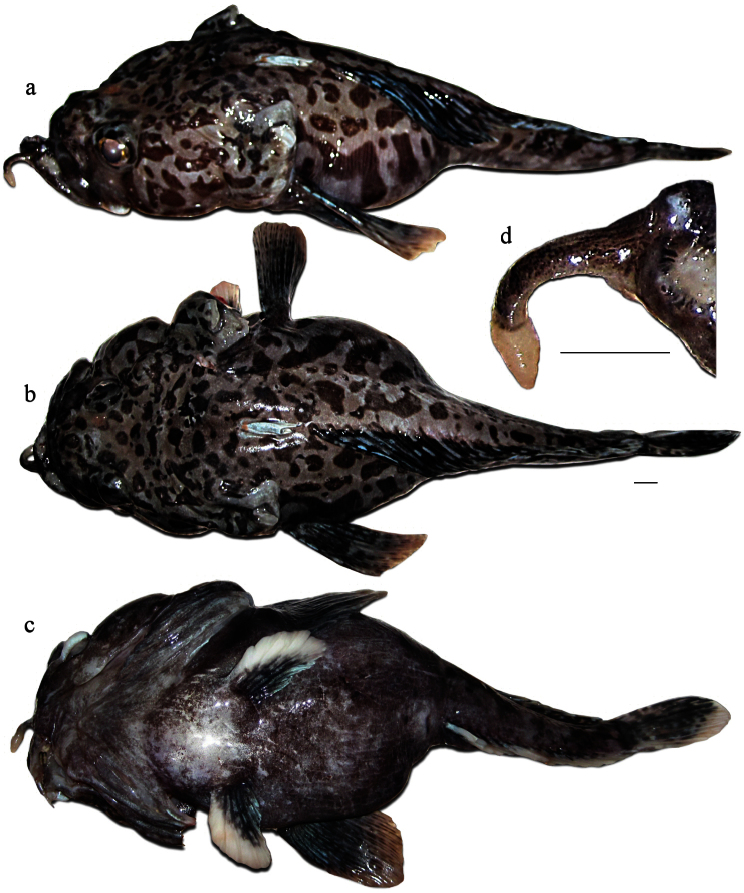
*Pogonophryne neyelovi* sp. n., freshly caught specimen (not preserved), 305 mm TL, 253 mm SL: **a** lateral view **b** dorsal view **c** ventral view **d** mental barbel (dorsolateral view). Scale bars 10 mm. Photo by Yu. Korzun. Scale bars 10 mm.

**Figure 5.**
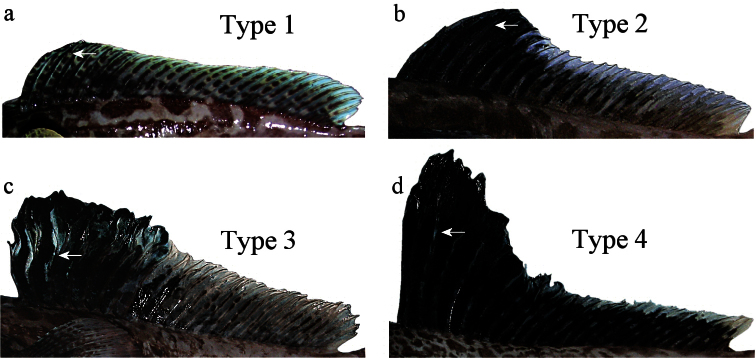
Four types of second dorsal fin shape in males of *Pogonophryne*. See text for explanations. Arrow indicates the point of primary dichotomic branching of anterior longest rays; **a** type 1: *Pogonophryne tronio*, holotype, 234 mm SL **b** type 2: *Pogonophryne* sp. 208 mm SL **c** type 3: *Pogonophryne neyelovi* sp. n., holotype **d** type 4: *Pogonophryne barsukovi*, 208 mm SL.

### Comparative remarks

*Pogonophryne neyelovi* sp. n. belongs to plunderfishes of the “*Pogonophryne mentella*” group that is characterized by the following characters: 1. the body and the head are covered with conspicuous dark markings; the spots are usually present on the top of the head, the nape and the back anterior to the first dorsal fin, spots on the top of the head varying in size and shape, mostly large, rounded, irregular or vermiculated, and usually sparsely distributed covering less than half the surface; 2. the anterior margin of the orbit is rounded, the eye entirely filling the orbit anteriorly; 3. the mental barbel is slender, tiny or stout and considerably varying in length (8–30% SL), its terminal expansion (inconspicuous or prominent) being present; 4. the inerorbital distance is wide (over 6% SL); 5. ridges on the top of the head are not well developed; 6. the snout slopes gradually (45° or less from the horizontal) in the lateral view; 7. the tip of the lower jaw is pointed or rounded and considerably varying with regard to its projecting beyond the upper jaw.

The “*Pogonophryne mentella*” group is the most speciose within the genus and comprises 13 currently recognized species (*Pogonophryne mentella*, *Pogonophryne macropogon* Eakin, 1981, *Pogonophryne lanceobarbata* Eakin, 1987, *Pogonophryne ventrimaculata*, *Pogonophryne cerebropogon* Eakin & Eastman, 1998, *Pogonophryne fusca* Ba-lushkin & Eakin, 1998, *Pogonophryne orangiensis* Eakin & Balushkin, 1998, *Pogonophryne eakini* Balushkin, 1999, *Pogonophryne squamibarbata* Eakin & Balushkin, 2000, *Pogonophryne bellingshausenensis*, *Pogonophryne brevibarbata*, *Pogonophryne tronio*,and *Pogonophryne neyelovi*) and five previously unknown forms considered possible undescribed species, *Pogonophryne* sp. C, D, E, F, and G (Shandikov 2013).

*Pogonophryne neyelovi* sp. n. belongs to the four species of the “short-barbeled subgroup” of the “*Pogonophryne mentella*” group characterized by the mental barbel not exceeding 13% SL. This subgroup also includes one shallow-water species, *Pogonophryne ventrimaculata*, known from the Weddell Sea and the Cosmonauts Sea, and two recently described deep-water species, *Pogonophryne brevibarbata* and *Pogonophryne tronio* that are sympatric with *Pogonophryne neyelovi* and captured from the same locality in the deep Ross Sea.

*Pogonophryne neyelovi* sp. n. clearly differs from *Pogonophryne ventrimaculata*, *Pogonophryne brevibarbata* and *P tronio* in having a very short (less than 1/3 of mental barbel length) and poorly developed ovaloid terminal expansion composed of bicolored, light and brownish, scale-like mostly bluntly palmate processes with transverse overall orientation (Figs 1d, 2b, 3d, 4d). In *Pogonophryne ventrimaculata* (Fig. 2d) the terminal expansion is well developed, bushy, stout and long (1/2 to 2/3 of mental barbel length), composed of light, mostly slender and tapered processes. In *Pogonophryne brevibarbata* it is tubiform and less developed than in *Pogonophryne ventrimaculata* but still long (more than 1/2 of mental barbel length), composed of light slender processes united mainly in longitudinally or obliquely oriented folds and rosettes (Fig. 2c). In contrast, in *Pogonophryne tronio* (Fig. 2a) the terminal expansion of the mental barbel is weakly developed, tapering and short (of about the same length as in *Pogonophryne neyelovi* sp. n.).

*Pogonophryne neyelovi* sp. n. further differs in having a very high second dorsal fin in male, its depth about 25% SL (*vs*. 15–21% SL in *Pogonophryne ventrimaculata*, 21% SL in *Pogonophryne brevibarbata*, and 16–18% SL in *Pogonophryne tronio*).

With regard to the shape of the dorsal fin, a short digression should be given here. There can be distinguished four types of the shape of the second dorsal fin in males of *Pogonophryne* (Fig. 5). Type 1(“*Pogonophryne tronio* type”, Fig. 5a): the fin is low (its height is up to 20% SL), slightly raised anteriorly, with a straight (not evidently concave) dorsal profile and without an anterior lobe; this type is known in six species (*Pogonophryne macropogon*, *Pogonophryne lanceobarbata*, *Pogonophryne cerebropogon*, *Pogonophryne squamibarbata* (?), *Pogonophryne bellingshausenensis*, and *Pogonophryne tronio*). Type 2 (“*Pogonophryne brevibarbata* type”, Fig. 5b): the fin is moderately high (its height is about 21–23% SL), definitely raised in its anterior part, with a concave or ladder-shaped dorsal profile, and commonly with a more or less prominent anterior lobe; this type is known in three species (*Pogonophryne ventrimaculata*, *Pogonophryne eakini*, *Pogonophryne brevibarbata*) and four undescribed forms (*Pogonophryne* sp. C, D, E and F) (Shandikov 2013). Type 3 (“*Pogonophryne neyelovi* type”, Fig. 5c): the fin is high (its height is about 25% SL), greatly raised in its anterior part, with an almost straight (not clearly concave) and gradually raised dorsal profile and without a prominent anterior lobe; this type is known in only one species, *Pogonophryne neyelovi* sp. n. Type 4 (“*Pogonophryne barsukovi* type”): the fin is very high (its height is 25–35% SL), considerably raised in the anterior part, with a clearly concave or ladder-shaped dorsal profile and a prominent anterior lobe; this type is found in five species (*Pogonophryne scotti* Regan, 1914, *Pogonophryne marmorata* Norman, 1938, *Pogonophryne barsukovi* Andriashev, 1967, *Pogonophryne immaculata* Eakin, 1981, *Pogonophryne stewarti* Eakin, Eastman & Near, 2009) and in one undescribed form (*Pogonophryne* sp. B) (Shandikov 2013). We could not classified the following seven species and one undescribed form as they have been known only by juveniles or females: *Pogonophryne permitini*, *Pogonophryne mentella*, *Pogonophryne albipinna* Eakin, 1981, *Pogonophryne platypogon* Eakin, 1988, *Pogonophryne dewitti* Eakin, 1988, *Pogonophryne fusca*, *Pogonophryne orangiensis* and *Pogonophryne* sp. A in Shandikov (2013).

Thus, as shown above, the shape of the second dorsal fin in the new species is rather peculiar (type 3, Fig. 5c) and possesses sinuous anterior rays that begin branching at about the mid-point of its length (*vs*. straight rays that begin branching at about the upper one forth of the ray in three other species in comparison). In contrast to males of *Pogonophryne brevibarbata* and *Pogonophryne ventrimaculata* (type 2, Fig. 5b) the male *Pogonophryne neyelovi* has neither a concave dorsal profile nor a prominent anterior elevated lobe in the second dorsal fin. In contrast to males of *Pogonophryne tronio* (type 1, Fig. 5a) it shows a significant difference in height of the anterior and posterior parts of the second dorsal fin (height of the posterior part of the second dorsal fin is 57% of maximum height of the second dorsal fin *vs*. 81–91% in *Pogonophryne tronio*).

The new species also differs from *Pogonophryne ventrimaculata* in lacking evident dark sharp spots and markings on the bases of the pectoral fins, the ventral parts of the head, breast and belly and in lacking any evident oblique dark striations on the second dorsal fin as well as in having uniformly pale pinkish anal and pelvic fins in adult individuals (*vs*. dark-light vertically bicolored or striped ones). It can be further distinguished from the latter in having more numerous total vertebrae (38 *vs*. 36–37). The new species has probably a deeper bathymetric distribution (700–1,390 m *vs*. 247–423 m).

The new species is morphologically closer to sympatric *Pogonophryne tronio* but it differs form the latter, besides the characters mentioned above, in having a more prominent lower jaw (teeth on the symphysis of the lower jaw are seen with the mouth closed *vs*. completely unseen in *Pogonophryne tronio*) and by the peculiarities of the coloration: in having an almost black second dorsal fin in the male (similar to *Pogonophryne brevibarbata*) rather than a sharply striped or mottled fin as in *Pogonophryne tronio*. In addition it may be noted that a recently post-spawning *Pogonophryne neyelovi* possesses remarkably fleshy and wide pelvic fins (up to 91% as wide as long) being similar in this regard to both sexes in post-spawning *Pogonophryne tronio* (81–90). Although the taxonomic validity of this character (probably nuptial) is still unclear in *Pogonophryne*, it was not so prominent in adult specimens of the other species with narrower pelvic fins (e.g. 52–60% as wide as long in *Pogonophryne brevibarbata*; Shandikov et al. 2013).

*Pogonophryne neyelovi* sp. n. further differs from *Pogonophryne brevibarbata* in having almost uniformly light pinkish anal and pelvic fins (similar to *Pogonophryne tronio*) rather than bicolored ones with a dark brownish basal part and a whitish distal part.

### Comparison material

The following 19 currently recognized species and seven undescribed forms of *Pogonophryne* were examined.

*Pogonophryne albipinna* (whitefin plunderfish): holotype (LACM 11353-1), juvenile, 37.5 mm SL [illustrated in Eakin (1981a: figs 3, 4)].

*Pogonophryne barsukovi* (stubbeard plunderfish): 5 non-type specimens (uncatalogued), males and females, 188–222 mm SL [listed and illustrated in Shandikov (2013: figs 4b, 5)].

*Pogonophryne bellingshausenensis* (spotless nape plunderfish): holotype (UAB-BS03-172A), male, 187 mm SL [illustrated in Eakin et al. (2008: figs 1−3)].

*Pogonophryne brevibarbata* (shortbeard plunderfish): holotype (ZIN 54969), male, 219 mm SL; 1 paratype (ZIN 54970), female, 254 mm SL; 1 non-type specimen (MNKhNU R298), female, 262 mm SL [illustrated in Shandikov et al. (2013 : figs 6−9)].

*Pogonophryne cerebropogon* (brainbeard plunderfish): holotype, male, 250 mm SL (USNM 345594) [illustrated in Eakin and Eastman (1998: figs 1, 2)].

*Pogonophryne dewitti* (DeWitt’s plunderfish): holotype (LACM 10485-3), juvenile, 55 mm SL [illustrated in Eakin (1988a: figs 1, 2)].

*Pogonophryne eakini* (Eakin’s plunderfish): holotype (ZIN 51706), male, 194 mm SL [illustrated in Balushkin (1999: figs 1, 2)].

*Pogonophryne fusca* (dusky plunderfish): holotype (ZMH 892-1986), female, 138 mm SL [illustrated in Balushkin and Eakin (1998: figs 1−3)].

*Pogonophryne immaculata* (spotless plunderfish): holotype (USNM 218370), female, 200 mm SL; 7 non-type specimens (NMNZ: P.37784, P.41416, P.37783, P.42633, P.43714, P.43715, P.43722), males and females, 188–209 mm SL [illustrated in Eakin et al. (2009: fig. 3)]; 1 non-type specimen (uncatalogued), female, 202 mm SL [illustrated in Shandikov (2013: fig. 1)].

*Pogonophryne lanceobarbata* (lancebeard plunderfish): holotype (ZMH 79−1985), female, 125 mm SL; 9 paratypes (ZMH: 48−1984, 52−1984, 78−1985, 85−1985, 96−1985, 110−1985, 189−1985), males and females, 61–194 mm SL [illustrated in Eakin (1987: figs 1, 2)].

*Pogonophryne macropogon* (greatbeard plunderfish): holotype (LACM 11402-4), male, 274 mm SL [illustrated in Eakin (1981a: figs 1, 2)]; 1 non-type specimen (ZMN 58−1991), female, 262 mm SL [listed in Eakin and Eastman (1998)]; 1 non-type specimen (uncatalogued), female, 235 mm SL [listed in Shandikov (2013)].

*Pogonophryne marmorata* (marbled plunderfish) (syn. *Pogonophryne minor* Balushkin & Spodareva, 2013): 9 non-type specimens, males and females, 45.6–167 mm SL [listed and illustrated in Eakin et al. (2001) and Shandikov (2013: fig. 4a)]; 1 specimen (holotype of *Pogonophryne minor*, ZIN 55237), female, 79.6 mm SL [illustrated in Balushkin and Spodareva (2013: figs 1–3)].

*Pogonophryne orangiensis* (orangebeard plunderfish): holotype (ZMH 42−1985), female, 164 mm SL, 3 paratypes (ZMH 111−1985, 123−1985; ZIN 51346), juveniles, 63–106 mm SL [illustrated in Eakin and Balushkin (1998: figs 1, 2)].

*Pogonophryne platypogon* (flatbeard plunderfish): holotype (SAIAB 25503), juvenile, 61 mm SL [illustrated in Eakin (1988b: fig. 1)]; 1 non-type specimen (uncatalogued), female, 141 mm SL [listed in Shandikov (2013)].

*Pogonophryne scotti* (saddleback plunderfish): 16 non-type specimens (uncatalogued), males and females, 157–200 mm SL [listed and illustrated in Shandikov (2013: figs 3a, b)].

*Pogonophryne squamibarbata* (scalebeard plunderfish): holotype, male, 147 mm SL (ZMH 65−1991) [illustrated in Eakin and Balushkin (2000: figs 1, 2)].

*Pogonophryne stewarti* (whipbeard plunderfish): holotype (NMNZ P.42288), male, 196 mm SL; 1 paratype (YPM 21196), male, 193 mm SL [illustrated in Eakin et al. (2009: fig. 1)].

*Pogonophryne tronio* (turquoise plunderfish): holotype (MNKhNU R295), male, 234 mm SL; 1 paratype (MNKhNU R296), female, 260 mm SL; 1 non-type specimen (MNKhNU R297), male, 230 mm SL [illustrated in Shandikov et al. (2013: figs 1−5) and Shandikov (2013: fig. 9)].

*Pogonophryne ventrimaculata* (spotbelly plunderfish): holotype (ZMH 46−1985), female, 214 mm SL; 4 paratypes (ZMH 93−1985, 115−1985, 190−1985), males and females, 115–214 mm SL [illustrated in Eakin (1987: figs 3, 4)]; 2 non-type specimens (uncatalogued), females, 141–222 mm SL [listed and illustrated in Shandikov (2013: fig. 6)].

Other comparative material of seven undescribed *Pogonophryne* species is listed and illustrated in Shandikov (2013: figs 2, 3c, d, 10−14).

### Key to the short-barbeled species of the “*Pogonophryne mentella*” group of the genus *Pogonophryne*

**Table:** 

1a	Ventral surface of head and abdomen with large, conspicuous dark spots. Mental barbel stout and with prominent long, bushy terminal expansion (50–66% of barbel length)	*Pogonophryne ventrimaculata*
1b	Ventral surface of head and abdomen without dark spots. Mental barbel thin, its terminal expansion short or long (18–68% of barbel length) but barely wider than stem or weakly developed	2
2a	Terminal expansion of mental barbel relatively long (1/2–2/3 of barbel length), tubiform and pointed terminally	*Pogonophryne brevibarbata*
2b	Terminal expansion of mental barbel very short (less than 1/3 of barbel length), ovaloid or weakly developed	3
3a	Terminal expansion of mental barbel ovaloid or hop-shaped, composed of scale-like, mostly bluntly palmate (resembling a cat’s paw) light brownish processes. Second dorsal fin in mature males black in anterior part and high (about 25% SL), with anterior rays sinuous distally	*Pogonophryne neyelovi* sp. n.
3b	Terminal expansion weakly developed, with flattened tip, composed of slightly tapered, fingerlike processes with wide, flattened bases and terminally with brown and light scale-like and leaf-like mostly serrated processes or/and low folds bearing prominent serrated leaf-like processes. Second dorsal fin in males darkly striped and mottled and relatively low (about 15–18% SL), with straight rays not sinuous distally	*Pogonophryne tronio*

## Supplementary Material

XML Treatment for Pogonophryne neyelovi
